# Novel *LMX1B* mutation in familial nail-patella syndrome with variable expression of open angle glaucoma

**Published:** 2007-04-27

**Authors:** Elena Millá, Imma Hernan, María José Gamundi, Maria Martínez-Gimeno, Miguel Carballo

**Affiliations:** 1 Departamento de Oftalmología, Hospital Clínic de Barcelona, Barcelona, Spain; 2 Servicio de Laboratorio, Hospital de Terrassa, Terrassa, Spain

## Abstract

**Purpose:**

To describe the genetic and clinical findings in a large Spanish pedigree with nail-patella syndrome (NPS) and to investigate the expressivity of open angle glaucoma (OAG) in the family members.

**Methods:**

All individuals underwent a complete ophthalmologic examination, including optical coherence tomography (OCT) of the optic disc and peripapillary region and ultrasound pachymetry. Screening for mutations in the *LMX1B* gene was performed by denaturing gradient gel electrophoresis and direct genomic sequencing analysis.

**Results:**

Ten family members had NPS, seven with varying degrees of ocular hypertension (OHT). Only one of these had advanced OAG. The others showed high pachymetry values and OCT retinal nerve fiber layer (RNFL) thickness above the normal values. Screening for mutations in the exonic and flanking sequences of the *LMX1B* gene showed a deletion of one G (289delG) within the coding sequence of exon 3 at codon 97, resulting in a frame shift that creates a premature stop at codon 105 (E97fsX105), predicting a truncated protein. This mutation was present in all NPS patients and absent in the unaffected family members.

**Conclusions:**

A novel mutation in the homeobox transcription factor *LMX1B* causes NPS in a family with variable expressivity of the syndrome, including OAG. The pathogenic mechanism resulting from the mutation is presumably haploinsufficiency rather than a dominant negative effect, which would explain the clinical variability in this family. All NPS OHT patients had considerably thick corneas and RNFL.

## Introduction

Nail-patella syndrome (NPS; OMIM 161200), also known as onychoosteodysplasia, Turner-Kieser syndrome, or Fong disease is a rare heterogeneous, autosomal-dominant pleiotropic disorder characterized by skeletal abnormalities such as nail dysplasia and hypoplastic patellae, renal disease, and open-angle glaucoma (OAG) [1-3]. More than 60 mutations in the *LMX1B* gene have been associated with NPS, which has a high intra- and interfamily degree of heterogeneity [4,5].

The identification of the causative gene for this syndrome, the LIM-homeodomain transcription factor *LMX1B*, led to investigation of the phenotype and molecular pathogenesis in different target organs in NPS patients [6-8]. The LMX-homeodomain proteins are a family of transcription factors frequently involved in pattern formation during embryonic development [9,10]. The *LMX1B* gene encodes a transcription factor protein that contains two zinc-binding LIM domains (A and B) at the NH_2_-terminus and one homeodomain in the middle, which is responsible for the binding to DNA [11-13]. Most of the mutations reported lead to the absence or inactivation of the homeodomain, resulting in a protein unable to recognize its target genes [14,15].

The LMX1B transcription factor protein is involved in normal patterning of the dorsoventral axis of the embryo during development [9,10] and early morphogenesis of the glomerular basement membrane [16]. Molecular studies and genetic immunochemical experiments carried out in the lmx1b^-/-^ mouse model for NPS showed the involvement of lmx1b in the transcription regulation of different alpha chains of type IV collagen in renal podocytes [17,18]. The defect in collagen fibrillogenesis is also seen in the cornea of lmx1b^-/-^ mutant mice, thus supporting the role of *LMX1B* in collagen regulation. These collagen defects in the eye could be responsible for the presence of glaucoma in NPS patients [19].

NPS is caused by a mutation in the *LMX1B* gene that presumably leads to loss of function of the encoding transcription factor, and the clinical phenotype is related to the pleiotropic effect of LMX1B during development [20,21]. It has been suggested that as the *LMX1B* gene encodes a LIM homeodomain transcription factor, which contributes to transcriptional regulation of glomerular membrane collagen expression by podocytes, both renal and ocular malformations could result from the same alteration during embryogenesis.

Many articles have cited the presence of OAG or ocular hypertension (OHT) as one of the features of NPS but the clinical details of this type of glaucoma have never been fully described. Recently, there has been an important advance in glaucoma diagnostic tools with the appearance of new imaging devices of the optic nerve head (ONH) and retinal nerve fiber layer (RNFL), such as optical coherence tomography (OCT) [22]. Moreover, the role of pachymetry in the differential diagnosis of OHT and glaucoma is progressively gaining more importance [23] prior to initiating expensive hypotensive treatments that can cause discomfort and side effects in these patients.

Our study had two aims: (1) to screen a seven-generation NPS Spanish family for mutations in the *LMX1B* gene; and (2) to characterize the clinical phenotype of their OAG or OHT by performing new structural optic disc examinations such as OCT.

## Methods

### Patients

Prior to their inclusion in this study, all participants were informed of its objectives. A total of 18 family members (12 women and 6 men) ranging in age from 7 to 90 years were analyzed. All gave their informed consent to participate in the study, which adhered to the tenets of the Declaration of Helsinki. Affected and unaffected members of a NPS Spanish family underwent a complete ophthalmologic examination and DNA genetic analysis. During the physical examination, some patients mentioned they were receiving topical glaucoma medications. After the initial examination, all patients were scheduled for several follow-up visits over three years. One patient, (IV-1), died a few months after the initial visit before undergoing other tests, such as pachymetry or OCT.

### Molecular genetics

Blood samples were drawn for DNA analysis. Genomic DNA was prepared from peripheral blood lymphocytes using QIAmp DNA blood mini kit (Qiagen, Valencia, CA). The coding region of the *LMX1B* (NM_00231) gene was amplified using primers located in the flanking intron region. Further amplification of exon 3 of *LMX1B* was carried out using 5'-CTG GGA GGG ACT TCT GAG CA-3' as the forward primer and 5'-CTC CAG GAC ACC CCA GCA AC-3' as the reverse primer. A "CG clamp", consisting of a sequence of 40 CG nucleotides, was included in the 5' sequence of the forward primer, 5'-CCT CCA GGA CAC CCC AGG AA-3', to provide better resolution in the denaturing gradient gel electrophoresis (DGGE) analysis [24]. Polymerase chain reaction (PCR) was performed in a 50 μl volume of buffer (20 mM Tris-HCl, pH 8.55; 16 mM (NH)_2_SO_4_; 1.5 mM MgCl_2_; 150 μg/ml BSA) containing 200-500 ng of human genomic DNA, 25 picomols of each primer, 10 nanomols of each deoxyribonucleoside triphosphate, and 1.5 units of Taq polymerase (Ecotaq). Incubation was performed for 40 cycles consisting of 1 min at 94 °C, 1 min at 50 °C, and 1 min at 72 °C; this was followed by 1 min at 94 °C and 5 min at 72 °C. Electrophoresis of 8 μl of final PCR reaction volume was performed on 1.5% agarose gel to test the amplification reaction. For DNA sequencing, PCR products were purified using Qiaquick columns (Qiagen). DNA sequencing was carried out using the OpenGene automated DNA sequencing system from Visible Genetics and Thermo Sequenase Cy5.5 Dye Terminator Cycle Sequencing kit (Amersham Pharmacia Biotech, Barcelona, Spain). The sequencing primers were the same as those used for the PCR reaction.

### Eye examination

The personal and family medical history of each patient was critical for determining which individual was affected and for establishing the inheritance pattern. Details about other systemic NPS anomalies were obtained from the each participant's medical chart. A comparison of the severity of systemic manifestations between the family members was performed to establish a spectrum of systemic involvement.

Best corrected visual acuity (BCVA) was tested with Snellen charts, and ocular refraction was evaluated with an autorefractometer. Biomicroscopy of the anterior segment was performed. Tonometry was done with a Goldmann applanation tonometer, using the mean of three consecutive tonometry readings for each eye. Gonioscopy with a Goldmann three-mirror lens and funduscopy for the observation of the ONH and peripapillary region under direct examination with a 78 D Volkmann lens completed the slit-lamp exam.

### Ancillary examinations

All cases underwent computerized perimetry with two types of perimeters to confirm the accuracy of the data. We used a 750-Humphrey field analyzer II following a SITA Standard strategy and an Octopus 101 perimeter, TOP strategy. We used a size III stimulus and a white-on-white test for both devices.

Monoscopic ONH and peripapillary RNFL digital pictures were taken with a Topcon nonmydriatic retinal camera (Topcon model TRC-NW6S, Topcon, Tokyo, Japan).

Ultrasound pachymetry was performed under topical anesthesia with an Ocuscan RxP pachymeter (Alcon Laboratories Inc., Irvine, CA) that provides six central readings and calculates the mean value. The intraocular pressure (IOP) is then manually introduced in each case and the machine automatically provides a new IOP value corrected for the pachymetry measurements (Herndon Formula).

Optical coherence tomography was performed with OCT version 3 (Stratus OCT, Carl Zeiss Meditec Inc., Dublin, CA), which is a high-resolution cross-sectional imaging technique that allows in vivo measurements of the RNFL (described further in reference [25]). All subjects received pupillary dilatation with 0.5% tropicamide.

Patients underwent three different protocols: fast RNFL thickness, fast ONH, and fast macular thickness measurements. The studied parameters in each protocol (average thickness, superior average thickness, inferior average thickness, vertically integrated rim area, horizontally integrated rim width, cup/disc area ratio, inferior outer macula, and temporal outer macula) were the most relevant for the glaucoma diagnosis according to the study by Medeiros; as all had receiver operating characteristic (ROC) curves above 0.75 except the disc area that was included in our study for demographic purposes [22].

In all protocols only images with a quality above 7 were accepted. Exams were repeated until this level was reached.

## Results

### Genetic analysis

**Figure 1:**
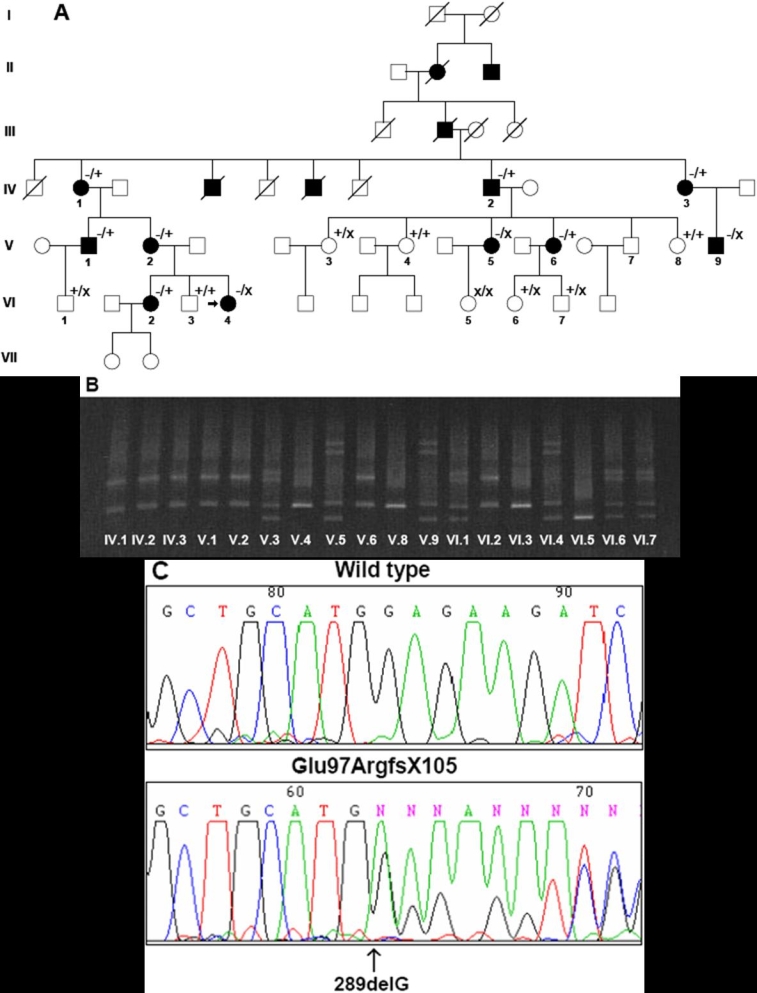
Genetic results of the NPS family. **A**: Pedigree of a Spanish family with nail-patella syndrome (NPS). Filled squares and circles denote NPS-affected males and females, respectively. Open squares and circles represent males and females without NPS. A diagonal line through the symbols marks a deceased individual. An arrow indicates the index patient (VI-4). The following symbols were used: wild-type allele (+)2889delG allele (-), and 372A>G allele (x), correspond to a, and allele, respectively. **B**: Denaturing gradient gel electrophoresis (DGGE) of the PCR fragment of exon 3. The different electrophoretic profiles correspond to carriers of the following: 289delG/WT (IV-1, IV-2, IV-3, V-1, V-2, V-6, and VI-2); WT/372A>G (V-3, VI-1, VI-6, VI-7); 289delG/372A gt G (V-5, V-9, VI-4); WT/WT (V-4, V-8, VI-3) and 372A>G/372A>G (VI-5). WT=wild type. **C**: DNA genomic sequencing of exon 3 of *LMX1B* showing the 289delG mutation (left) and a control DNA (right).

We screened for mutations in all of the exonic and flanking sequences of *LMX1B*: (NM_002307) in the index case of an NPS family. A DGGE alteration pattern was detected in exon 3 of this index patient but absent in 56 unrelated controls. Direct genomic sequencing revealed the deletion of one G (289delG) within the coding sequence of exon 3 at codon 97 of *LMX1B* (Figure 1). This nucleotide deletion changes the open reading frame of the gene, creating a premature stop at codon 105 (E97fsX105), presumably giving rise to a truncated protein. Both DGGE analysis and sequencing of exon 3 were carried out in all members of this family. Analysis of the family revealed that several members carried a novel nucleotide change 372 A>G that generates the silent mutation Glu124Glu (data not shown). Because mutations 289delG and 372>G in *LMXB1* segregate separately, as deduced by DGGE and sequencing analysis, both mutations would be in separate chromosomes (different alleles). It has been hypothesized that the severity of the NPS may also be related to the second copy of *LMXB1* gene [3]. In this large family we cosegregated both alleles (containing the NPS and the silent mutation), but no evidence supporting the hypothesis of a second allele interaction could be found.

**Figure 2:**
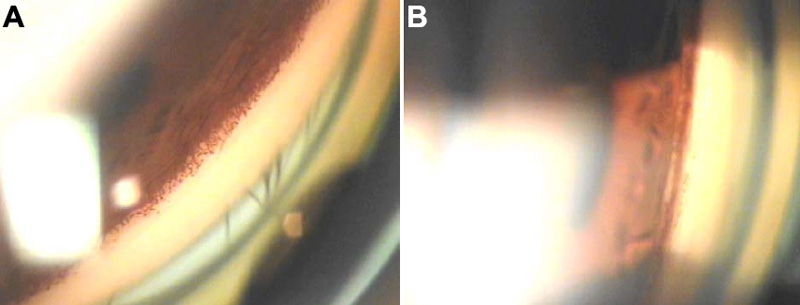
Gonioscopy of NPS patients. **A**: Color photograph taken during gonioscopy of multiple iris processes in patient V-6. **B**: Color photograph of a highly pigmented anterior chamber angle of patient V-9.

Ten family members had NPS with varying degrees of systemic involvement (Table 1), although one NPS member died (IV-1) during follow-up. The inheritance pattern was autosomal dominant with complete penetrance but variable expressivity (Figure 1). Onychodysplasia (Figure 2) and patellar hypoplasia were the only signs present in all the patients.

**Table 1:** Systemic phenotype of NPS family.

**Patient**	**Age**	**Sex**	**Nail dysplasia**	**Hypoplastic patellae**	**Other skeletal abnormalities**	**Colon nephropathy**	**Anomalies**	**OAG or OHT**
IV-1	86	F	+	+	+	+	-	+
IV-2	72	M	+	+	+	-	-	+
IV-3	67	F	+	+	+	-	+	+
V-1	60	M	+	+	+	-	+	+
V-2	56	F	+	+	+	-	-	-
V-5	46	F	+	+	+	-	-	+
V-6	41	F	+	+	+	+	-	+
V-9	29	M	+	+	-	-	-	+
VI-2	32	F	+	+	+	-	-	-
VI-4	28	F	+	+	+	+	+	-

Systemic anomalies of all nail-patella syndrome (NPS) patients evaluated in this study. The following terms were used: F refers to female, M refers to male, OAG is open angle glaucoma, and OHT is ocular hypertension.

Nephropathy (glomerulonephritis) and colon disease (spasmodic colitis) appeared in only a few members. Different skeletal abnormalities were observed in some members of the family: Patients V-5 and VI-4 showed hip subluxation, and patient VI-4 also had generalized joint hyperextensibility, particularly in the fingers. Some patients described generalized muscular pains (V-5, VI-2, VI-4) and chronic fatigue (V-5). The severity of the systemic involvement of NPS was unrelated to patient age, with the index patient being not only the youngest but also one of the most seriously affected.

### Ophthalmologic features

Seven of the NPS patients reported having OAG were treated by their ophthalmologists with one or two topical hypotensive drugs. Five of these complained of ocular side effects such as conjunctival hyperemia, itching, iris hyperpigmentation, and eyelash growth. These are well known common side effects of such hypotensive agents (Table 2).

**Table 2:** Opththalmologic features of NPS patients.

**Case**	**LMX1B haplotype**	**VA OD**	**VA OS**	**IOP OD**	**IOP OS**	**PACH OD**	**PACH OS**	**C IOP OD**	**C IOP OS**	**C/D OD**	**C/D OS**
IV-1	-/+	LP	HM	30	30	-	-	-	-	0.9	0.9
IV-2	-/+	0.4	0.3	26	24	581	591	23	20	0.3	0.3
IV-3	-/+	0.5	0.4	23	25	569	568	21	23	0.3	0.3
V-1	-/+	1	1	25	23	592	585	21	20	0.8	0.55
V-2	-/+	1	1	17	18	597	592	13	14	0.1	0.1
V-5	-/x	0.8	0.6	25	23	555	564	24	22	0.4	0.6
V-6	-/+	1	1	26	24	584	586	23	20	0.6	0.7
V-9	-/x	1	1	23	24	659	644	16	17	0.6	0.6
VI-2	-/+	1	1	18	19	596	590	14	15	0.1	0.1
VI-4	-/x	1	1	18	20	646	636	11	13	0.1	0.1

Ophthalmologic features of all nail-patella syndrome (NPS) patients with either open angle glaucoma or ocular hypertension. The following abbreviations were used: visual acuity (VA), right eye (OD), left eye (OS), light perception (LP), hand motion (HM), baseline intraocular pressure (IOP; in mmHg), pachymetry (PACH; in μm), pachymetry corrected IOP (C IOP), and cup/disc ratio (C/D). The following symbols were used in the *LMX1B* haplotype column: wild-type allele (+), 2889delG allele (-), and 372A>G allele (x).

BCVA was severely diminished in the eldest patient due to diabetic retinopathy with macular edema (IV-1). Two patients with cataracts (IV-2, IV-3) underwent uneventful bilateral phacoemulsification, and their BCVA was unremarkable in the postoperative period. A 48-year-old patient (V-5) presented with mild lens opacities that caused a certain degree of visual loss, suggesting the presence of congenital cataracts. The refractive status of all patients was within the normal range (data not shown).

Subjects receiving topical hypotensive medications were asked to stop their medications for a wash-out period of three to four weeks in order to establish a baseline intraocular pressure. Gonioscopy revealed the presence of multiple iris processes (Figure 2A) in the majority of the OAG patients and a highly pigmented trabeculum was also quite common, markedly so in one case (V-9; Figure 2B). No dysplastic changes or presence of abnormal vessels were noticed. All had wide, open anterior chambers and angles measured more than 45°, except in one case of 30°in both eyes (V-5).

One patient (V-1) presented with vertical saucerization in his right ONH and bilateral optic disc asymmetry, defined by a difference greater than 0.2 with left eye cupping. The eldest patient (IV-1) presented with a cup/disc ratio of 0.9, bayoneting of vessels and severe pallor, suggesting advanced glaucoma neuropathy.

Ultrasound pachymetry displayed high readings in all NPS patients in contrast with other non-NPS family members who had mean pachymetry readings of 530±3 μm.

Computerized perimetry was unremarkable in seven patients. Two (IV-2, IV-3) had diffuse decreased sensitivity due to their senile cataracts and presented with normal visual field testings after their cataract extraction. Patient V-5 had an initial inferior arcuate scotomata in both eyes, more marked in her right eye and mild diffuse loss due to congenital lens opacity (Octopus data in decibels (dB): MD 3.7; LV 9.9, OD/MD 2.2, LV 6.4 OS, and Humphrey perimeter data in dB: MD -5.14, DSM 3.57, OD/MD -2.40, DSM 2.09 OS). Patient IV-1 could not perform this test due to her severe low vision.

### Stratus optical coherence tomography data

**Figure 3:**
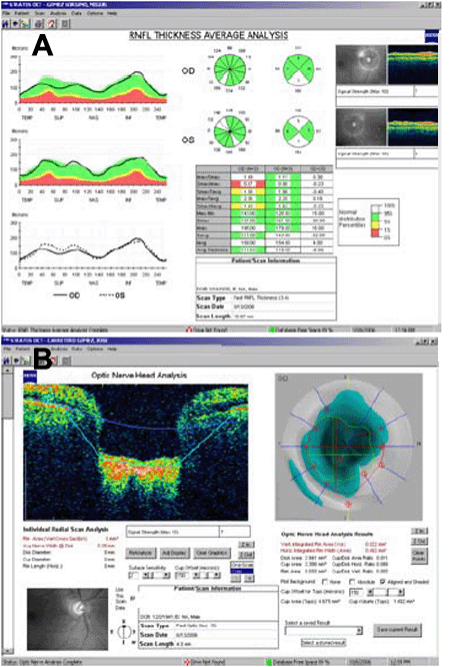
Optical coherence tomography of patients IV-2 and V-1. **A**: Optical coherence tomography (OCT) diagram for patient IV-2 in which above-normal retinal nerve fiber layer (RNFL) thickness measurements are expressed in white in the pie chart. Green sectors are within the normal range. **B**: OCT Fast optic nerve head protocol of patient V-1, confirming cup enlargement.

The Fast RNFL protocol showed extraordinary high thickness measurements through consecutive OCT tests (Table 3), showing that the peripapillary nerve fiber layer was thicker in these patients than in the normal population (data from 350 controls collected on the hard disk of the OCT machine). Five NPS patients (four with OHT and one normotensive) presented with mean thickness measurements in the "white zone" of the OCT plot (Figure 3A).

**Table 3:** Stratus optical coherence tomography data of nail-patella syndrome patients.

**Case**	**Eye**	**Fast RNFL protocol**			**Fast ONH protocol**						**Fast macula protocol**	
		**AT**	**ST**	**IT**	**VIRA**	**HIRW**	**Disc area**	**Cup area**	**C/D ratio**	**Rim area**	**IOM**	**TOM**
IV-2	OD	111.03	111	158	0.311	1.687	2.504	0.735	0.294	1.769	219	216
	OS	119.53	143.03	154	0.383	1.866	3.094	0.943	0.305	2.151	243	226
IV-3	OD	94.24	127	113	0.169	1.327	2.403	1.009	0.42	1.394	187	188
	OS	107.8	129	128	0.289	1.625	2.606	0.772	0.296	1.836	237	233
V-1	OD	94.29	134	106	0.022	0.492	2.941	2.386	0.811	0.555	205	209
	OS	99.52	136	134	0.142	1.584	3.735	2.486	0.666	1.249	219	215
V-2	OD	98.88	121	138	0.984	1.923	2.254	0	0	2.254	213	209
	OS	100.78	117	150	1.085	2.096	2.033	0	0	2.033	218	219
V-5	OD	118.52	160	139	0.205	1.664	2.745	1.4	0.51	1.345	239	222
	OS	111.38	138	139	0.258	1.754	2.751	1.206	0.438	1.545	251	216
V-6	OD	106.65	134	139	0.32	1.807	2.813	1.056	0.375	1.757	226	235
	OS	106.77	130	139	0.211	1.495	2.337	1.206	0.516	1.131	224	231
VI-2	OD	113.29	158	146	0.995	2.234	2.939	0.304	0.103	2.635	226	217
	OS	119.88	174	155	1.583	2.668	3.5	0.537	0.153	2.963	230	216
V-9	OD	111.75	142	141	0.265	1.635	2.485	1.224	0.493	1.261	237	218
	OS	115.15	146	147	0.29	1.827	2.965	1.227	0.414	1.738	232	211
VI-4	OD	135.54	163	199	0.788	2.434	3.102	0.683	0.22	2.419	243	226
	OS	126.03	179	181	0.735	2.613	4.209	1.291	0.307	2.918	248	232

The measurement numbers in red show thickness measurements above the normal range; the rest of the boxes show normal measurements. The following abbreviations were used: retinal nerve fiber layer (RNFL), optic nerve head (ONH) average thickness (AT), superior average thickness (ST), inferior average thickness (IT), vertically integrated rim area (VIRA), horizontally integrated rim width (HIRW), cup/disk area ratio (C/D ratio), inferior outer macula (IOM), and temporal outer macula (TOM). All values are expressed in mm except VIRA (mm^3^) and HIRW (mm^2^).

The Fast ONH protocol provided measurements of optic disc and cup diameters, volumes, and ratios that were also unremarkable except in patient V-I (Figure 3B). This patient presented with a pathologic cup/disc (C/D) area ratio in his OD (0.8) and bilateral asymmetry above 0.2 (C/D ratio 0.6 OS).

The Fast macular thickness protocol provided completely normal results for all the patients.

### Glaucoma therapeutic approach

Patient IV-I had been previously diagnosed with severe OAG based on tonometry and optic disc aspect and was under treatment with two drugs until she died.

Patients IV-2 and IV-3 were diagnosed with OHT or preperimetric OAG, and their topical hypotensive drugs were stopped. They presented normal perimetry and OCT at follow-up examinations after bilateral phacoemulsification.

Patient V-1 was diagnosed with glaucoma suspect upon optic nerve asymmetry between both eyes, confirmed by the C/D area ratio provided by OCT. He is being treated with one hypotensive drug (prostaglandin analog nightly).

Patient V-2 and her two daughters (VI-2, VI-4) were considered as non-OHT NPS patients.

Patient V-5 was deemed to have glaucoma based on the IOP readings and initial arcuate perimetry lesions, although the optic nerves were not apparently affected according to their aspect and the OCT. She is currently receiving hypotensive treatment with a prostaglandin analog and, as she is developing cataracts, will be scheduled for phacoemulsification.

Patients V-6 and V-9 were diagnosed with OHT because their IOPs were above 20 mmHg but less than 30 mmHg. Their ophthalmologic explorations were otherwise unremarkable. They currently remain without treatment.

## Discussion

We screened an index case from a large Spanish family with NPS for mutations in the *LMX1B* gene and found a novel nonsense mutation, 289delG, causing a premature stop at codon 105 (E97fsX105). The mutation is located in exon 3 of *LMX1B*, which encodes the COOH-terminal region of the LIM-A domain. Thus, in the putative-encoded mutant protein, the lack of the LIM-B domain and the homeodomain (HD) probably impede the DNA binding capacity of the protein.

To date, more than 60 point mutations, small deletions, or insertions have been reported, clustered in the LIM and HD domains of the *LMX1B* gene [14,15,20,21]. However, no mutations in NPS patients have been detected in the COOH-terminal third of the coding sequence of the *LMX1B* gene, suggesting that mutations in this region are not inactivating [15]. Furthermore, transfection studies performed with NPS mutants showed no significant dominant-negative effect on the function of the wild-type *LMX1B* [21,26]. These findings support haploinsufficiency as the mechanism underlying pathogenesis of NPS [20,21,27].

Mutations in *LMX1B* associated with NPS result in skeletal defects. All family members in this study who carried the E97fsX105 mutation showed nail dysplasia and hypoplastic patellae (Table 1), and most of them also had other skeletal abnormalities. These common features were present with almost complete penetrance in the family. Some typical characteristics were present at an early age, with members of the family being able to predict who were carriers of the disease. Indeed, their predictions agreed with our results concerning the carriers of the mutation in the *LMX1B* gene. However, renal, gastrointestinal, or ophthalmic involvement was more variable within the family.

The expression of *LMX1B* in the kidney occurs exclusively in podocytes [18]. Studies carried out in *lmx1b* knockout mice that mimic NPS showed an important impairment of the glomerular basement membrane with expression of the α3 and α4 chains of collagen IV and severe reduction of podocin [18,19]. Importantly, lmx1b is also necessary for normal development of multiple tissues in the anterior segment of the murine eye [19] Abnormalities in collagen fibrils in the corneal stroma of *lmx1b* knockout mice have been observed. The abnormal expression of collagen in homozygous *lmx1b* knockout mice may explain the split in the glomerular basement membrane in the kidney or the eye abnormalities [18,19] possibly related with glaucoma. Surprisingly, *lmx1b*^+/-^ mice do not show an abnormal phenotype, indicating differences in the *lmx1b* gene doses compared to humans. Recent immunohistological studies in NPS patients with severe glomerular disease suggest a possible regulation of type III collagen by *LMX1B*, while the homozygous mutations of *LMX1B* do not appear to dramatically affect the expression of type IV collagen chains and podocin in NPS patients.

Several well-known NPS studies have reported cosegregation of OAG and NPS as a result of a pleiotropic effect of the *LMX1B* gene in different families [1-3,14]. None of these studies described the glaucomatous features in detail. Some ocular abnormalities have been described in the knockout mouse model [19] such as an abnormal keratocan expression in the cornea, abnormal collagen fibrillogenesis, and a reduction in the depth of the anterior chamber. The *LMX1B* gene is also expressed in the trabecular meshwork but abnormalities in this tissue were not analyzed due to the short life span of the knockout mouse. In addition, many other ocular anomalies have been found sporadically in NPS patients, including microcornea, sclerocornea, congenital cataracts, iris processes, and pigmentation of the inner margin of the iris (referred to as Lester's sign) [1], but none of these is a specific trait of this syndrome.

Sweeney et al. [5] provided data from 123 NPS patients, most of whom were visited at home. IOP was measured using a Tonopen® and patients with IOP readings higher than 21 mmHg were advised to seek an ophthalmologist for further investigation. The researchers reported a prevalence of glaucoma and OHT of 9.6% and 7.2%, respectively. No other additional information of note for a glaucoma diagnosis is available in their study. Bongers et al. [27] gave more reliable information. In their study, patients underwent gonioscopy and Humphrey visual field analysis. They found glaucoma and OHT in 35.3% of the patients and reported other ocular anomalies, such as corneal anomalies, iris pigmentation, pigment dispersion syndrome, and congenital cataracts. Lichter et al. [1] reported OAG in 13 of 33 families, some of whom had IOP higher than 30 mmHg and required filtration surgery. Recently, a large study by Mimiwati [2] found glaucoma in 11.1% of his NPS population (33% of cases over the age of 40 years) and OHT in 11.1%. They reported an average age at diagnosis of glaucoma in cases with apparently *LMX1B* causative mutations of 29.7 years, which is substantially below the expected age at diagnosis for most cases of primary OAG. The authors performed a comprehensive glaucoma screening that included IOP, automated perimetry, and stereo disc photographs on all available family members.

New imaging techniques, such as OCT, are becoming useful tools for the structural analysis of the optic disc and peripapillary region, target sites of glaucomatous damage. The most useful protocols are the Fast RNFL thickness and Fast ONH, and several OCT parameters are presently being used to evaluate the presence of glaucomatous optic disc damage [22].

This is the first report of OCT data in NPS patients with glaucoma or OHT. Surprisingly, all our study patients presented RNFL thickness measurements above normal. This indicates that there is no loss of peripapillary nerve fiber bundles and thus excludes the presence of glaucomatous optic neuropathy in our study population. These data correlated well with the aspect of the optic disc and the perimetric results, except in patient V-5, who presented arciform scotomata. This patient requires future perimetry testing after phacoemulsification, as the cataract could skew the perimetry results. One patient (V-1) presented with marked optic disc cupping according to the examiner's subjectivity; it was supported by the ONH OCT protocol.

Pachymetry-measured central corneal thickness has a significant effect on the clinical management of patients with glaucoma and glaucoma suspects [28]. In 1970, Ehlers [29] published a paper suggesting that corneal thickness, another biomechanical measurement of the eye, also affected IOP. Technology to measure corneal thickness has developed over time, and it was not until the publication of the Ocular Hypertension Treatment Study (OHTS) [23] in 2002 that central corneal thickness (CCT) was suggested to be an independent risk factor for progression to initial glaucoma damage among individuals with OHT.

A thin pachymetry reading under 530 μm is considered a risk factor for developing glaucoma in eyes with OHT. Recent general practice guidelines recommend performing routine pachymetry and applying a correction factor to the IOP measurements in order to avoid a confounding effect. An association has even been proposed between CCT and the elasticity of the lamina cribrosa. A very recent study [30] even suggested an inverse correlation between CCT and optic disc area. While thicker corneas may cause examiners to make a slight overestimation of true IOP, their thickness may also indicate the presence of a substantially smaller, and thus more robust, ONH. Conversely, individuals with thinner corneas, which may initiate a slight underestimation of true IOP, may also be those with larger and more deformable optic discs. Our study population corroborated this fact: All presented with thick corneas and thick RNFL measurements whereas non-NPS family members had "normal" CCTs. Whether thick CCT belongs to the constellation of NPS traits is still unknown and requires routine pachymetry in all these patients.

Our study population had OHT that fell within "normal" limits of IOP readings after the automated pachymetry corrections performed by the Ocuscan®, as they all had very thick corneas. According to the OHTS [23], they all have a low risk of developing OAG in the future. Moreover, their functional and structural tests were normal, and medical treatment was not required in these cases. It was actually withdrawn in several cases except those with suspected glaucoma. We do not know if any other NPS glaucoma cases described in the literature would have OHT or possible glaucoma after pachymetry IOP correction.

The role that iris processes play in the development of OHT is unknown, as they have classically been considered to be innocuous, unlike the presence of high trabecular pigmentation. Almost all our OHT and non-OHT NPS patients had a myriad of iris processes in their anterior chamber angles. This trait, therefore, seems to accompany the other NPS manifestations, as has been described before, and is independent of IOP.

In the family reported, some patients had OHT and kidney or colon abnormalities. The 30% proportion of the family who had gastrointestinal and renal abnormalities of NPS is similar to previously reported data. The presence of glaucoma or OHT has been estimated to be near 30% in other studies, but in this family 70% of the NPS patients had OHT, indicating that this is a significant feature in the family. OHT was not a constant sign and its severity did not depend on the age of the patient or the degree of systemic disease. The index patient in this family was one of the most affected individuals, with severe skeletal anomalies and nephropathy, though she did not have OHT.

We conclude that NPS shows a high inter- and intrafamily heterogeneity. The pathogenesis of OAG or OHT in these patients is still not well understood, and it shows no correlation with the severity of other systemic anomalies. OHT was not present in unaffected individuals, and it seems to be a genuine pleiotropic effect of the *LMX1B* mutation in this family.

Caution should be taken when diagnosing the presence of glaucoma in persons with NPS, and it should be corrected by pachymetry. OCT is a useful tool for the diagnosis of glaucomatous optic nerve and RNFL pathology, especially in those cases with unclear perimetric testing. We encourage other investigators to perform pachymetry and OCT in NPS patients in order to corroborate the presence of a higher thickness of the corneal and retinal nerve tissues. Surprisingly both factors could, in a certain way, be considered as "protective" ocular mechanisms against the presence of glaucomatous optic nerve damage. There is still a long way to go in the establishment of a correlation between glaucoma and other ocular manifestations of this syndrome, such as the presence of multiple iris processes. Ultrastructural studies are needed to establish whether both renal and ophthalmic anomalies belong to the same form of inheritable connective tissue disorder.
